# The Invisible Bond of Trauma: Examining Sibling Influence on Psychological Distress in Syrian Refugee Youth

**DOI:** 10.21203/rs.3.rs-9003440/v1

**Published:** 2026-05-19

**Authors:** Yusra Sannah, Liza Hinchey, Erin Williamson, Mahmood Sanad, Arash Javanbakht

**Affiliations:** Wayne State University; Wayne State University; Wayne State University; Wayne State University; Wayne State University

**Keywords:** Refugee, youth, sibling, anxiety, post-traumatic stress disorder (PTSD), trauma

## Abstract

**Background:**

As the global refugee crisis escalates, conflict continues to traumatize and displace an increasing number of children, exposing them to considerable psychological challenges. Although studies have found links in psychological distress among native-born, non-refugee siblings and other refugee family dyads, none have examined possible correlations among adolescent refugee siblings.

**Methods:**

We conducted a prospective, longitudinal analysis of Syrian refugee youth anxiety and post-traumatic stress disorder (PTSD)-like symptoms. Data was collected within one month of arrival to the U.S. and two years later. Correlation analyses of sibling dyads were performed, controlling for trauma exposure, to determine the independent influence of sibling symptom severity on mental health outcomes.

**Results:**

Upon resettlement, anxiety symptoms significantly predicted sibling anxiety (b = 1.47, p < 0.001), though no association emerged between sibling PTSD symptoms. Two years post-resettlement, neither PTSD nor anxiety symptom severity correlated among siblings.

**Conclusion:**

Coupling of anxiety in the immediate aftermath of forced migration may be more likely between siblings, whereas later anxiety may be more related to independent life and resettlement factors. Similarity in post-resettlement anxiety symptoms among siblings underscores shared experiences and the need for family-based interventions.

## Introduction

Siblings of children with mental illness (MI) are at increased risk for developing psychopathology due to both genetics and shared environment (Barnett & Hunter, 2012; Buist et al., 2019; van Sprang et al., 2023). A significant portion of a child’s environment is shaped by their familial relationships. Families with children who have mental health conditions tend to demonstrate more negative relationships between siblings and between parents and children, compared to families without MI (Ma et al., 2017). Still, the effects of having a sibling with MI extend beyond negative relationships to impact mental wellbeing. Available data indicates that 19% of siblings of children with MI demonstrated signs of depression and anxiety, 16% exhibited aggression, and 12% displayed defiant rebellious behavior (Barnett & Hunter, 2012). The correlated mental health of siblings not only exists in childhood but may also persist into adulthood. Siblings of adults with MI (depression, anxiety, psychotic, bipolar, and eating disorders) have demonstrated greater depression and anxiety symptoms compared to adults without a sibling with MI (Jayasinghe et al., 2023)--indicating a link between having a sibling with MI and increased risk of psychological distress across the lifetime. Beyond interpersonal relationship dynamics, additional mechanisms may underlie this sibling pattern. Recent longitudinal studies have revealed that an older sibling’s depression predicts depression in the younger sibling one year later, independent of gender or interpersonal closeness between siblings (Buist et al., 2019).

Childhood and adolescence represent particularly vulnerable developmental stages regarding these relational patterns. During youth, psychological development is strongly influenced by both sibling dynamics and family environment. Sibling support and identification can reduce externalizing problems, while problematic sibling behavior is linked to internalizing issues (Bank & Kahn, 1982; Branje et al., 2004). Indirect effects stem from disrupted family cohesion, imbalanced parental attention, or stigma associated with a sibling’s mental illness (Bank & Kahn, 1982; Branje et al., 2004; Smith et al., 2016). Shared environmental stressors, learned maladaptive behaviors, and genetic vulnerabilities also contribute to sibling relationships and mental health outcomes (Burt, 2009; Bussell et al., 1999; Johnson et al., 2001).

### Mental health of youth with trauma exposure

Prior studies of youth affected by trauma reveal a complex interplay between mental health and family dynamics. Parental mental health plays a significant role in shaping children’s long-term emotional well-being, particularly in families exposed to trauma. For instance, in families with a recently deceased child, maternal, but not paternal, symptoms of PTSD and prolonged grief disorder were directly associated with surviving siblings’ outcomes (Morris et al., 2016); however, analysis of sibling influence on mental health following trauma is limited.

Although mental health status may correlate among siblings when one child has a pre-existing mental health condition, it is unclear whether shared traumatic and adverse experiences lead to associated post-traumatic symptoms. Sibling relationships are increasingly recognized as a salient factor in shaping the impact of trauma on youth mental health. In refugee populations, for instance, the sibling relationship may become particularly crucial due to reduced external social support. War-traumatized refugee children report lower social support from friends compared to both native-born and non-refugee children (Peltonen et al., 2010). Exposure to military trauma has also been linked to heightened sibling rivalry and diminished friendship quality, both of which mediate relations between trauma and mental health symptoms (Peltonen et al., 2010). Conversely, sibling relationships may also serve as protective factors following trauma, with positive sibling interactions associated with reduced anxiety, depression, anger, and post-traumatic stress symptoms in children exposed to trauma (Perricone et al., 2014).

### Refugee youth mental health

The global refugee population continues to expand at an alarming rate. According to the United Nations High Commissioner for Refugees (UNHCR), the number of forcibly displaced people has been steadily rising over the past decade. By mid-2024, it is estimated that 122.6 million people were forcibly displaced due to conflict, violence, persecution, and human rights violations worldwide (UNHCR, 2024). This is the highest number ever recorded in history. Of all forcibly displaced people, children account for 40%–despite making up 30% of the world population (UNHCR, 2024). This global crisis underscores the urgent need for research that addresses the psychological condition of refugee children, potential influence of family relationships on mental health, and their psychological needs.

In addition to the psychological impact of war, migration and resettlement strain the mental wellbeing of refugee families. Studies of refugees indicate a high prevalence of PTSD, anxiety, depression, and behavioral problems (Betancourt et al., 2012; Bogic et al., 2015; Eruyar et al., 2018; Javanbakht et al., 2018; Reavell & Fazil, 2017). Pre-migration trauma, particularly exposure to violence and family member deaths, is significantly correlated with psychiatric symptoms, i.e., PTSD or anxiety (Hecker et al., 2018; Heptinstall et al., 2004; Hinchey, Chammaa, et al., 2025). Post-migration stressors, including insecure asylum status, family separation, financial difficulties, and social isolation, are associated with increased depression symptoms (Hecker et al., 2018; Heptinstall et al., 2004; Hinchey, Alahmad, Gorski, & Javanbakht, 2025; Hinchey, Alahmad, Gorski, Jenuwine, et al., 2025).

Refugee siblings share both: 1) genetic and epigenetic overlaps in vulnerability to trauma impact, and 2) traumatic experiences, and pre- and post- migration stressors. Still, each child also has a unique biological, psychological, and environmental milieu, contributing to risk and resilience. While exposure to war or natural disaster and forced migration may lead to more connected experiences following resettlement, siblings’ experiences also have potential to diverge. Greater insight into family-related predictive factors of refugee mental health, particularly during vulnerable childhood and adolescent stages, could improve our ability to accurately estimate risk, allocate resources, and support refugee families.

Previous work assessing the mental health of Syrian refugee children and their families found an elevated prevalence of anxiety and post-traumatic stress symptoms (Javanbakht et al., 2018). In many cases, symptoms either remained chronically high, or progressively worsened over time (Hinchey, Nashef, et al., 2023). Maternal symptom severity across multiple domains (i.e., anxiety, depression, and PTSD) was also associated with anxiety symptom severity in their children (Javanbakht et al., 2018). Similarly, married couples also demonstrate associated mental health symptoms (including PTSD, anxiety, and depression), likely due to shared trauma exposure and the impact of spousal stress (Khalil et al., 2023). To extend the body of research aimed toward understanding the influence of family relationships on psychiatric symptoms in refugee youth, the current study queried independent relations of youth mental health symptom severity on their sibling’s symptom severity in recently resettled Syrian refugee youth exposed to war trauma, forced migration to refugee camps, and resettlement in the US. Our specific aims were to: 1) assess correlation in anxiety and PTSD-related symptoms among siblings at the time of arrival in the U.S.; and 2) determine whether siblings’ symptoms correlated two years later.

We hypothesized that, similar to other previously assessed family dyads in this refugee population, anxiety and PTSD symptom severity would be correlated between siblings, especially in the context of shared refugee trauma and stress experiences. We further hypothesized that a stronger association would be observed in the siblings upon arrival in the US, which would lessen over time due to stabilization and differentiation of their environment (e.g., development of external social support; greater variation in life experiences). These findings may have implications for future interventions, particularly those that consider the broader familial context in promoting better mental health outcomes for refugee youth.

## Methods

### Study Design

To better understand how shared trauma, stress, and psychological distress in one refugee child may be associated with increased distress in a sibling, we analyzed Syrian and Iraqi war refugee sibling mental health scores obtained following migration and resettlement in the U.S in 2016. Follow-up visit data was collected from the same participants approximately 2 years later. Prior to resettlement, participants had lived in refugee camps for 2 years before U.S. entry.

### Participants and Procedures

Syrian and Iraqi refugees were recruited from two clinics operated by a community agency **[blinded]** that provides mandatory physical health screenings for recently arrived refugees. Upon completion of the primary health screening, families were given the opportunity to participate in a paid, voluntary research study. Children with parental consent were taken to a private room for an explanation of the study, consent process, and survey, conducted by linguistically and culturally fluent research assistants. All materials and questionnaires were made available in both Arabic and English, though all youth participants chose to complete them in English. Youth participants were included when willing and able to provide oral assent (ages 7–12) or written assent (ages 13–17), with parent/guardian providing written informed consent. Following initial data collection at time of arrival in the U.S. (n = 178), youth provided data approximately 2 years post-arrival (n = 193). Additional youth were recruited during Phase 2 to account for elevated attrition in refugee populations **[blinded]**. All procedures were approved by the **[blinded]** Institutional Review Board.

### Measures

Participant characteristics were collected via a demographic questionnaire. Trauma exposure was assessed using the Life Events Checklist for DSM-5 (LEC-5), which identifies participants’ exposure to sixteen distinct potentially traumatic events (PTEs) (Weathers et al., 2013). The data collected focused on instances of direct exposure and excluded indirect exposure (i.e., hearing about a PTE). Specifically, the number of “Happened to me” responses across the 16 items was summed to generate both a total trauma exposure score and scores for validated trauma subtypes (Gray et al., 2004). The LEC-5 has demonstrated strong validity and reliability across cultures (Lima et al., 2016; Rzeszutek et al., 2018). Recently, trauma subtypes were found to be more informative than cumulative trauma, informing this analysis to control for trauma subtype with the same convention (Hinchey, Grasser, et al., 2023).

Psychological distress was assessed using two self-report screening tools: the Screen for Child Anxiety Related Emotional Disorders (SCARED) and the PTSD Checklist for *DSM-5* (PCL-5). The SCARED questionnaire is a 41-item screening tool developed for children (ages eight to eighteen) to quantify severity of anxiety-like symptoms (Birmaher et al., 1999). Responses are reflected in a 3-point Likert scale covering domains of panic/somatic, separation anxiety, generalized anxiety, and social phobia based on the *DSM-IV* (Birmaher et al., 1999). The Arabic version has previously been evaluated, demonstrating satisfactory psychometric properties (Hariz et al., 2013). The reliability of this tool in the current study was high (α = 0.931). The PTSD Checklist for *DSM-5* (PCL-5) is a 20-item questionnaire used to measure the severity of participants’ PTSD-related symptoms. Previous reports validated this tool across languages (i.e., Arabic) and forms of trauma exposure (Ashbaugh et al., 2016; Bovin et al., 2016; Ibrahim et al., 2018). The PCL-5 demonstrated high reliability (α = 0.941).

### Data Analysis

Data analysis was performed using IBM SPSS (Version 30.0). Descriptive statistics were examined to assess outliers, distributional properties, missing data, and assumptions. Data cleaning procedures followed best practices (Kwak & Kim, 2017; Newman, 2014). Sibling pairs were randomly selected by first selecting one sibling pair per family, then randomly assigning one as the “participant” and the other as the “comparison sibling,” with all other siblings excluded. This process resulted in 50 sibling pairs in Phase 1 (N = 100) and 51 in Phase 2 (N = 102) after excluding pairs with missing symptom data. Model assumptions for linearity, homoscedasticity, and normality of residuals were met. Pairwise correlations indicated no issues of multi-collinearity (r>.8 for all). Pairwise correlations and t tests were inspected for possible demographic covariates (i.e., age, sex, country of origin, and ethnicity), none of which were significant. Age, sex, and trauma exposure were included as likely covariates based on prior literature (Breslau, 2002). Percentages of missing data in Phase 1 (PTSD: 34%, anxiety: 44%) were moderately high; multiple imputation using 8 imputations was used to generate analysis data. No data was missing at scale level for PTSD nor anxiety in Phase 2; therefore the original data set was used for analysis. Data was found to be missing completely at random (MCAR; Little’s x2 = 27.19). For trauma covariates, three continuous trauma subtype variables were computed by summing “yes” responses to LEC-5 items related to directly experiencing events within each subtype (see Table 1) **[blinded]**. Hierarchical linear regressions were used to examine whether youth symptom severity predicted sibling symptom severity, controlling for age and sex. Separate models were run for anxiety and PTSD. Trauma exposure variables were entered as covariates in a second step to assess the unique contribution of youth symptoms. Analyses were conducted separately for Phase 1 (using imputed data) and Phase 2 (using original dataset).

## Results

### Descriptive Statistics:

Demographic characteristics are outlined in Table 1. Within the 102 participants included in this study, 49% were female and mean age was 12.76 years (*SD* = 2.91). Most participants were of Arab ethnicity (97.1%), and the remaining population was Kurdish (2.9%). On average participants reported experiencing 1.11 cumulative trauma exposures (*SD* = 2.16); 27.5%, 13.7%, and 22.5% reported exposure to accident/injury, victimization, and death threat trauma subtypes, respectively. Average severity of post-traumatic stress (*M* = 21.06, *SD* = 21.07) and anxiety (*M* = 24.07, *SD* = 14.46) symptoms were moderate.

### Main Analyses:

#### Phase 1

For the Phase 1 model predicting PTSD-related symptoms, the PTSD symptom predictor variable and covariates of age, sex, and trauma subtype exposure were regressed on the dependent variable of sibling PTSD symptom severity (see Table 2). No significant association emerged between child PTSD symptoms and their sibling PTSD symptoms at Phase 1. The anxiety Phase 1 model was subsequently tested, entering sex, age, and trauma subtype exposure as covariates and child anxiety symptoms as a predictor of sibling anxiety. Here, child anxiety symptoms were significantly associated with their sibling’s anxiety (b = 1.47, SE = .26, t = 5.58, p < .001).

#### Phase 2

For the Phase 2 model observing PTSD-related symptoms, the PTSD symptom predictor variable and covariates of age, sex, and trauma subtype exposure were regressed on the dependent variable of the severity of sibling PTSD symptoms (Table 2). At Phase 2, there was no significant relation between child PTSD symptoms and their sibling PTSD symptoms. The anxiety Phase 2 model was then tested, entering sex, age, and trauma subtypes as covariates and child anxiety symptoms as a sibling anxiety predictor. Contrary to findings from the Phase 1 anxiety model, no significant relationship emerged between child anxiety and their sibling’s anxiety symptoms.

## Discussion

In this study, we assessed anxiety and PTSD symptom severity correlations among refugee youth siblings upon resettlement in the U.S. and two years post. We controlled trauma exposure to determine the independent influence of sibling symptoms on mental health outcomes. Initially, shared experiences of war, migration, and resettlement appeared to affect siblings similarly, as youth anxiety symptom severity significantly correlated with sibling anxiety. In line with our hypothesis that any initial associations would weaken over time, this correlation did not persist two years post-resettlement. In contrast to anxiety, youth PTSD symptoms did not correlate with their sibling’s PTSD symptoms at either time point.

Our first wave of data was collected within the first month of refugee families’ arrival in the host community. At that time, siblings are more likely carrying shared traumatic experiences, and importantly, shared stresses of transition from home through migration and resettlement in the host country. Later in resettlement, siblings’ lives and experiences may diverge and each will have their individual “normal life” stressors, which may help explain the absence of a sustained association between siblings’ anxiety symptoms across the resettlement period. As children adapt to their new environments, they may become less reliant on siblings and family members than they were prior to migration, including reduced dependence on siblings for social support. Additionally, as environmental stress decreases with the removal of acute threats associated with war, there may be less need to seek refuge within the family system to buffer against a high-threat environment, resulting in weaker within-family coupling of anxiety symptoms over time.

In both phases, PTSD symptom severity was not correlated between siblings. This is important to be seen in the context that more youth showed much higher anxiety-related symptoms than PTSD symptoms **[blinded]**. In other words, PTSD symptoms were low to begin with, and fewer participants reached the diagnostic criteria for PTSD as compared to anxiety disorder. Furthermore, while there is a higher likelihood of shared stressors through the average two years of migration from home country to the US, traumatic experiences might be more varied among family members. Another possible explanation is that PTSD may be more strongly shaped by individual subjective appraisals rather than by shared post-migration circumstances, resulting in various symptoms even within the same family. In addition, PTSD development can be non-linear, with delayed onset or fluctuating symptoms vary across individuals depending how the person may respond to trauma. Early post-arrival resettlement stressors may also preferentially produce shared anxiety across siblings, whereas PTSD symptoms may be less synchronized during a period of acute transition. Adjusting for trauma subtype may have reduced observed sibling associations by accounting for shared exposure-related variance, and the limited sibling-dyad sample size may have reduced statistical power to detect modest correlations in PTSD severity.

Patterns of sibling mental health concordance in refugee youth may reflect shared pre-migration trauma followed by increasing divergence in post-migration experiences over time. Although prior work in non-refugee samples suggests that associations between siblings’ mental health may extend into adulthood, even as siblings’ environments become increasingly differentiated over time (Jayasinghe et al., 2023), our results point to a distinct pattern for refugee youth siblings: early concordance in anxiety may reflect shared war- and migration-related stressors, whereas the weakening of sibling symptom similarity over time may reflect increasing divergence in individual post-migration experiences. Differences in schooling, peer networks, language acquisition, and stressor exposure during resettlement may contribute to increasingly distinct mental health trajectories within families.

The concordance in youth sibling anxiety aligns with previous work showing within-family similarities in psychological distress among Syrian refugees, including associations between maternal symptoms across anxiety, depression, and PTSD and children’s anxiety (Javanbakht et al., 2018), as well as concordance in PTSD, anxiety, and depression among married couples, potentially reflecting shared trauma exposure and spousal stress (Khalil et al., 2023). This result also aligns with broader literature suggesting that siblings’ mental health is often interrelated, with studies linking an index youth’s mental illness to elevated distress and poorer adjustment in other siblings, potentially through shared family environments and sibling dynamics (Barnett & Hunter, 2012; Buist et al., 2019; van Sprang et al., 2023; Jayasinghe et al., 2023). However, most prior studies have focused on non-refugee and clinically identified samples, whereas our study examined refugee adolescent sibling dyads longitudinally during resettlement.

Several factors may explain why sibling stress symptoms affect a child, including shared biological vulnerabilities, experiences, and environmental influences. Direct environmental influences include sibling support and sibling identification, whereby siblings emulate each other’s behaviors consciously or unconsciously (Bank & Kahn, 1982; Branje et al., 2004). Indirect influences arise from changes in family dynamics, such as disrupted family cohesion, unbalanced parental attention, or social stigma related to a sibling’s mental illness. For instance, strong parental support and cohesion are protective, while weaker support correlates with greater psychological challenges (Fazel et al., 2012). When support by parents and friends are controlled, higher initial levels of sibling support correlated to lower initial levels of internalizing problem behaviors for both older and younger adolescents siblings ages eleven to fifteen–though this difference may not persist over time (Branje et al., 2004). Instead, internalizing behaviors appear more significantly impacted by a sibling’s internalizing behavior, rather than sibling support for both younger and older siblings (Branje et al., 2004). Similar studies with a focus on adolescent externalizing behaviors have controlled for parental and peer influences, finding that specifically older sibling externalizing problems may be a unique social risk factor for adolescent externalizing problems (such as delinquency and aggression), equal in strength to significant parents’ and friends’ risk factors (Defoe et al., 2013). These findings expose areas for future research to clarify the contributions of sibling behaviors, support, and broader social factors.

This study has several limitations. Firstly, restrictions on refugee entry to the U.S. during the period of data collection limited the sample size of this investigation. Secondly, self-report screening assessments were collected verbally in the family setting, which may have led to under-reporting symptoms due to cultural or familial perceptions of mental health. When compared to parental assessments, refugee children reported fewer internalizing symptoms (more prominent among girls) and fewer externalizing symptoms (more prominent among boys) (Montgomery, 2008). Thus, self-reported data may not represent the full extent of the mental health symptoms facing refugee children. Thirdly, no direct measurement of shared environment or sibling influence was collected/assessed. While this study suggests that shared environment and/or sibling influence might explain early anxiety correlations, we did not directly measure these factors. However, this same cohort has previously shown correlation between all factors of maternal stress and those of children **[blinded]**. Other unexamined variables, such as sibling relationship quality, possible caregiving role, or sibling order, could play a role. Future research should explore other trauma-affected populations, control parental and/or peer influences, and investigate the unique impact of siblings in various cultural contexts. Comparing refugee adolescents with those of migrant populations without war trauma may clarify the role of trauma versus migration in shaping mental health trajectories (Betancourt et al., 2017; Spaas et al., 2022).

## Conclusion and Implications

This work is among few longitudinal studies that examine mental health symptom correlation among refugee youth siblings across resettlement. Findings suggest that early, post-arrival anxiety symptoms may more likely align within sibling dyads, potentially reflecting shared experiences or vulnerabilities. Anxiety later in resettlement may be more likely influenced by diverse individual and ecological factors. Results support targeted screening to identify those at higher risk for anxiety burden, especially when one family member shows higher signs of stress. Additionally, given this finding and previous findings on correlation of stress among refugee mothers and children, family oriented and group-based trauma informed interventions could be helpful especially in low resource contexts.

## Figures and Tables

**Table 1:** Demographic characteristics and descriptive statistics of sample

Age	Range	*M*	*SD*
	7–19	12.76	2.91
Female, %		49.0	
Country of origin, %			
Iraq		2.9	
Syria		97.1	
Ethnicity, %			
Arab		97.1	
Kurdish		2.9	
Accident/Injury	0–4	.34	.67
Victimization	0–3	.20	.56
Death threat	0–6	.48	1.13
Cumulative trauma	0–14	1.11	2.16
PTSD Symptoms	0–98	21.06	21.07
Anxiety Symptoms	0–57	24.07	14.46

*Note*. Ranges refer to the range of sample, not possible range of scores on measures (n = 102). LEC-5 items included in each subtype category are as follows: 1) accident/injury (items 1–4 and 12); 2) victimization (items 6, 8, and 9); and 3) death threat (items 5, 7, 10, 11, and 13–16; **[blinded]**).

**Table 2:** Effects of cumulative trauma and trauma subtypes on psychological symptoms

Model	Independent Variable	Sibling PTSD symptoms		Sibling Anxiety symptoms	
		*β*	*p*	*β*	*p*
Phase 1					
	(constant)	8.84	.585	5.89	.421
	Age	.32	.847	−1.56*	.008
	Sex (female)	−24.05	.091	−5.51	.200
	Victimization	−3.69	.720	4.08	.523
	Death Threat	3.61	.336	16.64*	<.001
	Accident/Injury	2.64	.768	−27.40*	<.001
	Anxiety Symptoms	--	--	1.47*	<.001
	PTSD Symptoms	.26	.288	--	--
Phase 2					
	(constant)	.18	.987	12.08	.238
	Age	.82	.284	.49	.465
	Sex (female)	1.46	.731	− .28	.937
	Victimization	4.83	.319	6.36	.112
	Death Threat	3.38	.277	−3.27	.207
	Accident/Injury	7.1	.125	3.00	.436
	Anxiety Symptoms	--	--	.21	.114
	PTSD Symptoms	.15	.163	--	--
Phase 1 Model R^2^		.386		.913	
Phase 2 Model R^2^		.387		.182	
Phase 1 Model Adjusted R^2^		− .074*		.826	
Phase 2 Model Adjusted R^2^		.303		.071	

*Note*. Predictor variables are marked significant at the *p<.05 level. The R^2^ for significant models based on *F* statistics are noted with an asterisk.

## Data Availability

Wayne State University requires data use agreements to be drafted and approved prior to any data sharing. If investigators are interested in accessing data, agreements will need to be drafted and approved between institutions and investigators in order to protect these data. Therefore, data cannot be made publicly available at this time, however, data can be made available by request to the authors and institutional approval with an appropriate data use agreement.
